# Hemithorax Westermark Sign Secondary to Complete Pulmonary Artery Occlusion from Pulmonary Embolus

**DOI:** 10.5811/cpcem.2021.2.51043

**Published:** 2021-03-01

**Authors:** Michael Louthan, Daniel Ng

**Affiliations:** Riverside Community Hospital, Department of Emergency Medicine, Riverside, California

**Keywords:** Westermark, pulmonary embolism, radiology

## Abstract

**Case Presentation:**

We describe a complete right hemithorax Westermark sign found in a patient with a near-complete, right pulmonary artery trunk occlusion secondary to a pulmonary embolus.

**Discussion:**

We review the sensitivity and specificity of a Westermark sign in the identification of a pulmonary embolism, and how this aided us in managing our patient in the emergency department.

## CASE PRESENTATION

**Image 1:**
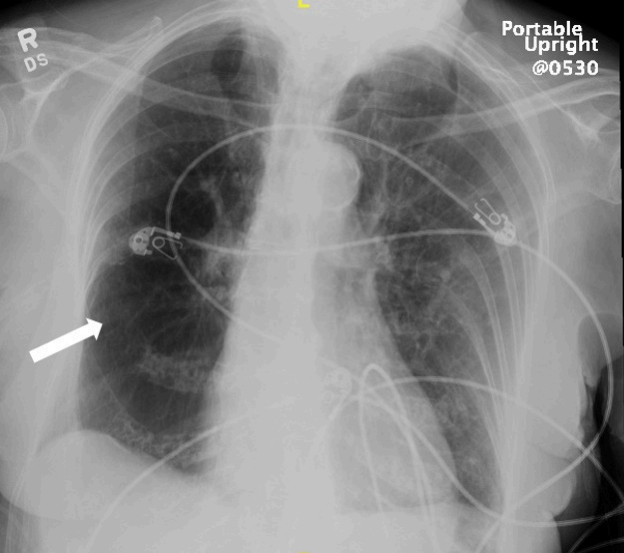
Chest radiograph. Image is notable for increased lucency within the right hemithorax (arrow).

**Image 2:**
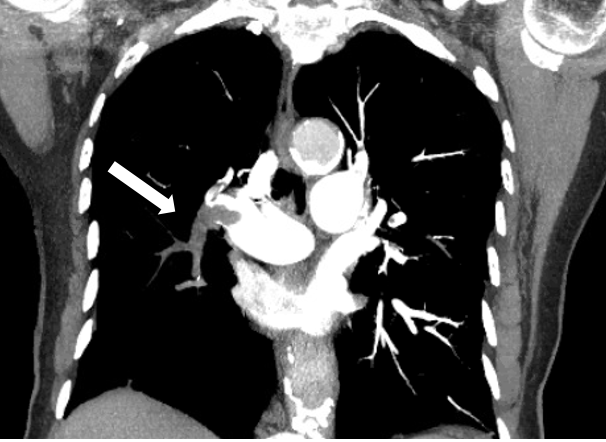
Coronal view of the computed tomography pulmonary angiogram. Image is notable for pulmonary embolus within right pulmonary artery trunk with almost complete occlusion of segmental branches (arrow).

**Image 3:**
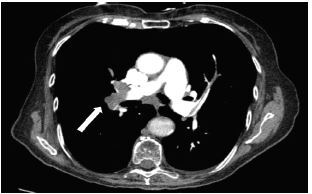
Transverse view of the computed tomography pulmonary angiogram. Image redemonstrates the almost-complete occlusion of the right pulmonary artery trunk with the pulmonary embolus (arrow).

An elderly female with a history of chronic obstructive pulmonary disease, recent left fibular fracture, and prior lung cancer now in remission presented to the emergency department secondary to dyspnea for six days. While she did report some decreased mobility, she would not be described as immobilized, as she was still able to care for herself independently. Her vital signs were as follows: temperature of 97.5º Fahrenheit; heart rate of 89 beats per minute; blood pressure of 171/74 millimeters of mercury; respiratory rate of 18 breaths per minute; and pulse oximetry of 88% oxygen on room air. Her hypoxemia corrected with supplemental oxygen via nasal cannula, albuterol/ipratropium nebulizer, and intravenous (IV) steroids. Chest radiograph noted an increased translucency within the right hemithorax, consistent with Westermark sign (Image 1). Further evaluation via computed tomography pulmonary angiogram was notable for a pulmonary embolus (PE) within the right pulmonary artery trunk extending into nearly all segmental and subsegmental branches (Images 2, 3). The patient was treated with IV heparin and admitted for further work-up.

## DISCUSSION

We describe a case of undifferentiated dyspnea that was found to have an impressive Westermark sign on chest radiograph due to a proximal and occlusive PE. First described in 1938, Westermark sign refers to an increased lucency in a portion of lung due to PE. This hyperlucency is due to proximal mechanical obstruction of blood flow leading to impaired vascularization and resultant oligemia of affected lung fields. Westermark sign has a 14% sensitivity and 92% specificity for PE identification.^2^,^3^ Although not diagnostic alone, Westermark sign can be helpful in pursuing the diagnosis of PE.

**CPC-EM Capsule**

What do we already know about this clinical entity?

*Westermark sign has low sensitivity but high specificity for pulmonary embolism identification.*

What is the major impact of the image(s)?

*Complete occlusion of a pulmonary artery trunk can lead to a hemithorax Westermark sign.*

How might this improve emergency medicine practice?

*When faced with increased translucency of a hemithorax, physicians must consider near-occlusion of a proximal pulmonary artery and treat accordingly.*

## References

[1] Westermark N (1938). On the roentgen diagnosis of lung embolism: brief review of the incidence, pathology and clinical symptoms of lung embolism. Acta Radiol.

[2] Worsley DF, Alavi A, Aronchick JM (1993). Chest radiographic findings in patients with acute pulmonary embolism: observations from the PIOPED study. Radiology.

[3] Stein PD, Beemath A, Matta F (2007). Clinical characteristics of patients with acute pulmonary embolism: data from PIOPED II. Am J Med.

